# A synchronized VUV light source based on high-order harmonic generation at FLASH

**DOI:** 10.1038/s41598-020-63019-2

**Published:** 2020-04-22

**Authors:** Elisa Appi, Christina C. Papadopoulou, Jose Louise Mapa, Nishad Wesavkar, Christoph Jusko, Philip Mosel, Skirmantas Ališauskas, Tino Lang, Christoph M. Heyl, Bastian Manschwetus, Maciej Brachmanski, Markus Braune, Hannes Lindenblatt, Florian Trost, Severin Meister, Patrizia Schoch, Rolf Treusch, Robert Moshammer, Ingmar Hartl, Uwe Morgner, Milutin Kovacev

**Affiliations:** 1 Institut für Quantenoptik, Leibniz Universität Hannover, Hannover, 30167 Germany; 2 Cluster of Excellence PhoenixD (Photonics, Optics, and Engineering – Innovation AcrossDisciplines), Hannover, 30167 Germany; 3 DESY, Hamburg, 22607 Germany; 4 EXC 2123/1 QuantumFrontiers, Hannover, 30167 Germany; 5 Helmholtz-Institut Jena, Jena, 07743 Germany; 6 Max-Planck-Institut für Kernphysik, Heidelberg, 69117 Germany

**Keywords:** High-harmonic generation, Atomic and molecular interactions with photons

## Abstract

Ultrafast measurements in the extreme ultraviolet (XUV) spectral region targeting femtosecond timescales rely until today on two complementary XUV laser sources: free electron lasers (FELs) and high-harmonic generation (HHG) based sources. The combination of these two source types was until recently not realized. The complementary properties of both sources including broad bandwidth, high pulse energy, narrowband tunability and femtosecond timing, open new opportunities for two-color pump-probe studies. Here we show first results from the commissioning of a high-harmonic beamline that is fully synchronized with the free-electron laser FLASH, installed at beamline FL26 with permanent end-station including a reaction microscope (REMI). An optical parametric amplifier synchronized with the FEL burst mode drives the HHG process. First commissioning tests including electron momentum measurements using REMI, demonstrate long-term stability of the HHG source over more than 14 hours. This realization of the combination of these light sources will open new opportunities for time-resolved studies targeting different science cases including core-level ionization dynamics or the electron dynamics during the transformation of a molecule within a chemical reaction probed on femtosecond timescales in the ultraviolet to soft X-ray spectral region.

## Introduction

**Figure 1:**
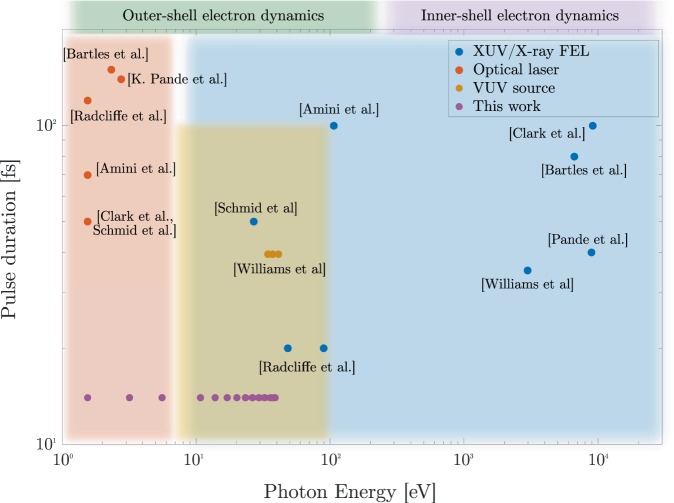
Pump-probe parameter ranges of selected experiments in which an external optical laser was used in combination with a XUV/X-ray FEL^35–41^. The pulse duration of pump and probe pulses is displayed together with the corresponding photon energy. Data labels and markers indicate references and instrument (blue: XUV/X-ray FELs, red: optical lasers including bulk-based frequency converted lasers, orange: VUV sources). The HHG-beamline described in this work is indicated in violet. Please note that the represented data are not intended to be exhaustive, but rather to give an overview on the available parameter spaces at FEL facilities.

Since 2005 free-electron laser (FEL) facilities provide extreme ultraviolet (XUV) to X-ray radiation with pulse energies in the microjoule to millijoule range and very short pulse durations in the order of 100 fs down to 1 fs^1–8^, enabling high X-ray peak intensities^9^. These sources facilitated groundbreaking advances in the understanding of light-matter interactions in many fields including condensed matter physics, protein structure determination or fundamental atomic physics^3^. A very large part of these experiments focus on unraveling the ultrafast processes when matter changes its internal configuration, for example the transformation of a molecule during a chemical reaction^10^ or phase changes in magnetic structures^11^. These studies are typically performed in a laser-FEL pump-probe scheme, where a pump pulse initiates the reaction in the sample and a probe pulse probes the state of the sample after a given time delay. Employing FELs in combination with modern femtosecond lasers as pump and/or probe while applying sophisticated jitter corrections, time resolutions below 100 fs (FWHM) are achievable at FEL facilities^12–16^. The central wavelength of the pump and probe pulses must be chosen with respect to the specific reaction under study. Today’s short wavelength FEL facilities cover wavelength ranges from 1 to 500 Å (see Fig. 1) and femtosecond lasers are nowadays also available over a large spectral range. Via frequency conversion of femtosecond laser pulses with nonlinear optical crystals a spectral range from the deep ultraviolet (DUV) to the THz can be covered^17–20^. An extension towards shorter wavelengths is possible by employing well-known high-harmonic generation (HHG) based approaches^21–23^. While the FEL FLASH can cover most of the produced HHG spectral range with high temporal resolution via split-and-delay units, a synchronized HHG source allows independent tuning of pump and probe central wavelength, for example combining a long wavelength VUV pump pulse with a soft X-ray probe pulse. In addition, the HHG source provides much broader spectral bandwidth than the FEL, which is beneficial for transient absorption experiments to cover multiple spectral resonances within one acquisition. This way, the combination of two powerful extreme ultraviolet femtosecond sources, FELs and HHG-based sources, can enable VUV-XUV pump-probe experiments, which are ideally suited for studying time-resolved light-matter interaction of small quantum systems after VUV excitation and/or ionization. They may allow for benchmark studies enhancing the basic principles of charge migration, charge transfer and electronic relaxation pathways of small molecules and might provide new insights into dynamics beyond the Born-Oppenheimer approximation in many-body systems.

Here we report on a HHG-based vacuum ultraviolet (VUV) source connected to the free-electron laser FLASH in Hamburg. This source provides femtosecond laser pulses covering a wavelength range from 30 to 120 nm, thus extending optical lasers available at FEL facilities into the VUV. The source is integrated in the FLASH beamline FL26^24^, which hosts a state-of-the-art reaction microscope^25^ (REMI) as a permanent end-station. The VUV source is characterized and employed for first proof-of-principle studies enabling the production and detection of Ar^+^ ions inside REMI, demonstrating the capability of this source for future VUV-XUV pump-probe studies.

## Methods

**Figure 2:**
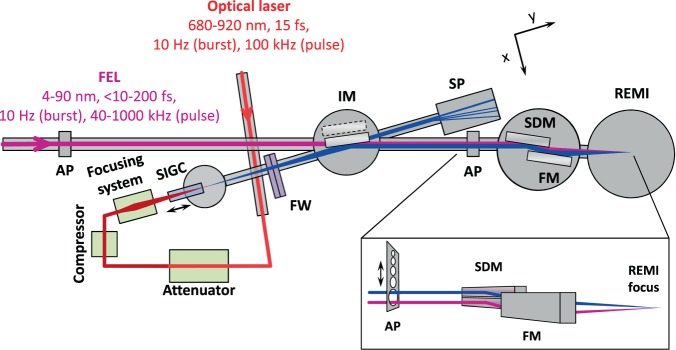
VUV source incorporated in the FEL beamline. SIGC: semi-infinite gas cell, FW: double filter wheel, IM: in-coupling mirror, AP: apertures array, SDM: split-and-delay mirror, FM: focusing ellipsoidal mirror, SP: spectrometer.

The generation of high harmonics by intense infrared femtosecond pulses in a gas medium is a well-known and established approach to provide coherent radiation in the VUV to XUV range. However, here we face the challenge that the source points of FEL and HHG are dislocated by about 80 meters. In order to allow for good focusing conditions at the REMI end-station, the optics design of the HHG beamline (see Fig. 2) needs to adapt to a virtual source point, which is accomplished by a specially designed in-coupling hyperboloid mirror. Another challenge towards the full integration of a HHG beamline is the synchronization. The generated harmonics naturally depend on the properties of the FLASH2 pump-probe laser system that allows generating a tunable spectrum of ultrashort harmonic pulses. The implementation of the optical laser system, which is synchronized with the FEL burst pulse pattern, is further detailed in the following subsections. Concerning the beamline vacuum requirements, the REMI end-station sets challenging conditions. The harmonic generation in a typical gas cell can reach pressures of a few mbar up to atmospheric pressure, but in our case the operational vacuum in REMI needs to be in the order of 10^−11^ mbar at the same time. Differential pumping stages have been designed to bridge the pressure difference up to 10 orders of magnitude from the HHG gas cell to the in-coupling mirror chamber in the FEL beamline. Finally, monitoring the HHG output is challenging due to space restrictions and the need to characterize not only the spectral distribution but also the available photon flux. We choose here an in-line approach with a transmission grating spectrometer and a calibrated large area photodiode, which is described in detail below.

### Optical pump-probe laser

The HHG process is driven by the FLASH2 pump-probe laser system^19^, which is based on optical parametric chirped pulse amplification (OPCPA). This technology allows for the generation of intense, high repetition rate and wavelength tunable ultrashort laser pulses. The laser system consists of two chirped pulse laser amplifiers (CPA), which deliver high repetition rate ultrashort pulses for the generation of broadband OPCPA seed pulses as well as a high average power pump. Both CPA’s are seeded by a common low noise ytterbium laser oscillator synchronized via balanced optical cross-correlation (BXC) to a length stabilized pulsed optical fiber synchronization system^26^. This combination allows for excellent synchronization between laser oscillator and optical link with an integrated timing jitter of <5 fs. Timing drifts in the amplifier system introduced by environmental changes are actively compensated below 6 fs rms^27^. Additional drifts of the OPCPA system are currently under investigation. The system is capable of matching the burst-mode timing structure of the FEL featuring 10 Hz pulse trains and intra-burst repetition rates up to 1 MHz within the maximum burst duration of 800 μs (typical operation mode: 100 kHz).

The laser pulses are transported in vacuum to the VUV experimental setup via a 40 m long relay-imaging transport beamline. We employ an active drift stabilization of the beam position and pointing before the beam enters the transport line, based on cameras and piezo actuated mirrors. This leads to measured pointing jitter at the setup of 8% rms of the beam diameter over 120 hours, similar to the performance of the laser before beam transport. In addition, the beam position is monitored before the coupling to the HHG gas cell, which could also be used for an active pointing feedback. The total time jitter after the beam transport is yet to be explored.

The compression of the laser pulses is realized close to the VUV setup by a pair of double chirped mirrors (DCMs) optimized to minimize higher order dispersion (Fig. 2). With 20 bounces on the DCM compressor mirrors a total negative dispersion of −1500 fs^2^ (−75 fs^2^ per bounce) is applied to reach the measured pulse duration of 15 fs (FWHM). For this short pulse duration, the central wavelength spans from 680 to 920 nm. In order to optimize the HHG efficiency the pulse energy can be attenuated via a half-waveplate and a wedged thin film polarizer pair. Additional quarter- and half-waveplate stacks are used to optimize the polarization of the HHG driving laser for linear polarization. Pulse energies of up to 225 μJ with 780 nm central wavelength at 100 kHz intra-burst repetition rate (peak-to-peak pulse energy stability <5% rms) are focused in the gas target of the VUV source in the experiments presented here. S-polarization is chosen in order to get the highest reflectivity on the mirrors used in the beamline.

### VUV beamline

The infrared OPCPA pulses are focused in a gas medium to drive high order harmonic generation of the fundamental wavelength. Since the single pulse energy is limited by the maximum output power and the high intra-burst repetition rate, the beam is focused down to a diameter of 72 μm in a tight focusing geometry, in order to achieve intensities above 1 × 10^14^ W/cm^2^ required for the HHG process. The focusing system consists of one defocusing and one focusing mirror (radii of curvature: 1000 mm and −600 mm respectively) separated by 165 mm at a small angle (≈5°). The effective focal length of the telescope is 56 cm. The residual astigmatism of the beam can be minimized by precise alignment of the system.

**Figure 3:**
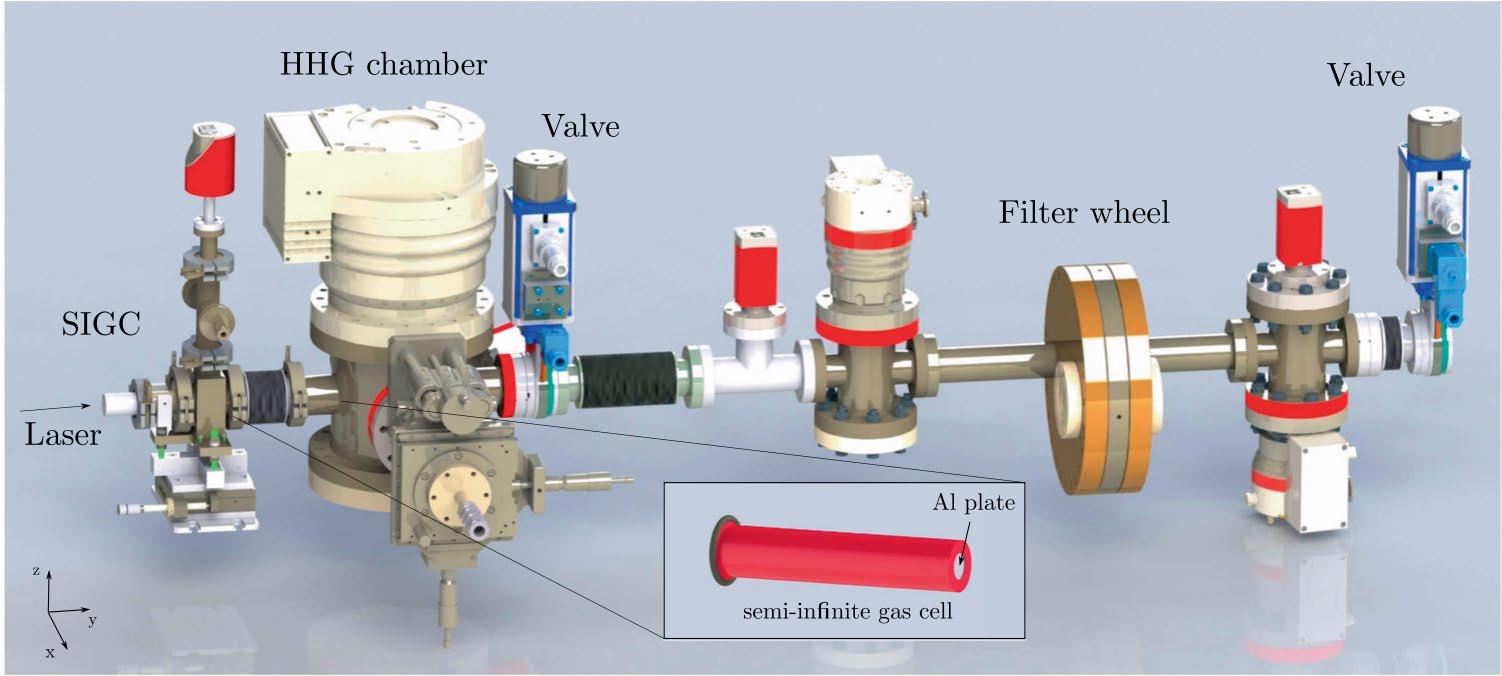
Scheme of the VUV line. The optical laser enters the SIGC from the left side through the fused silica window and drills a hole in the aluminium plate (shown in the inset). The generated radiation propagates from the HHG chamber towards the filter wheel.

The gas medium is contained in a semi-infinite gas cell (SIGC), which consists of a 30 cm tube with a 0.5 mm thick fused silica window and a 0.5 mm thick aluminium plate at the beam entrance and exit side respectively, shown in the inset of Fig. 3. The relatively long length of the tube ensures that the intensity on the window stays below the damage threshold. The focused laser beam drills a hole of <100 μm in the plate, which allows for the propagation of the radiation while maintaining the required pressure gradient between the gas cell and the beamline. The system, inserted and connected to the HHG chamber with a bellow (see Fig. 3), is placed on a linear translation stage (LT1, Thorlabs), which allows a ±2.5 cm movement along the beam path direction for the optimization of the distance between the beam focus and the Al plate.

To suppress the remaining infrared beam and/or to select a harmonic spectral window of interest, we use a rotatable double filter wheel. In total 11 filters can be installed in each wheel giving the possibility to use different materials and thicknesses. The combination of different filters allows for selecting specific photon energy ranges. So far no damage has been observed on the 0.1 μm thick aluminium filter used during the commissioning over several hours of irradiation.

**Figure 4:**
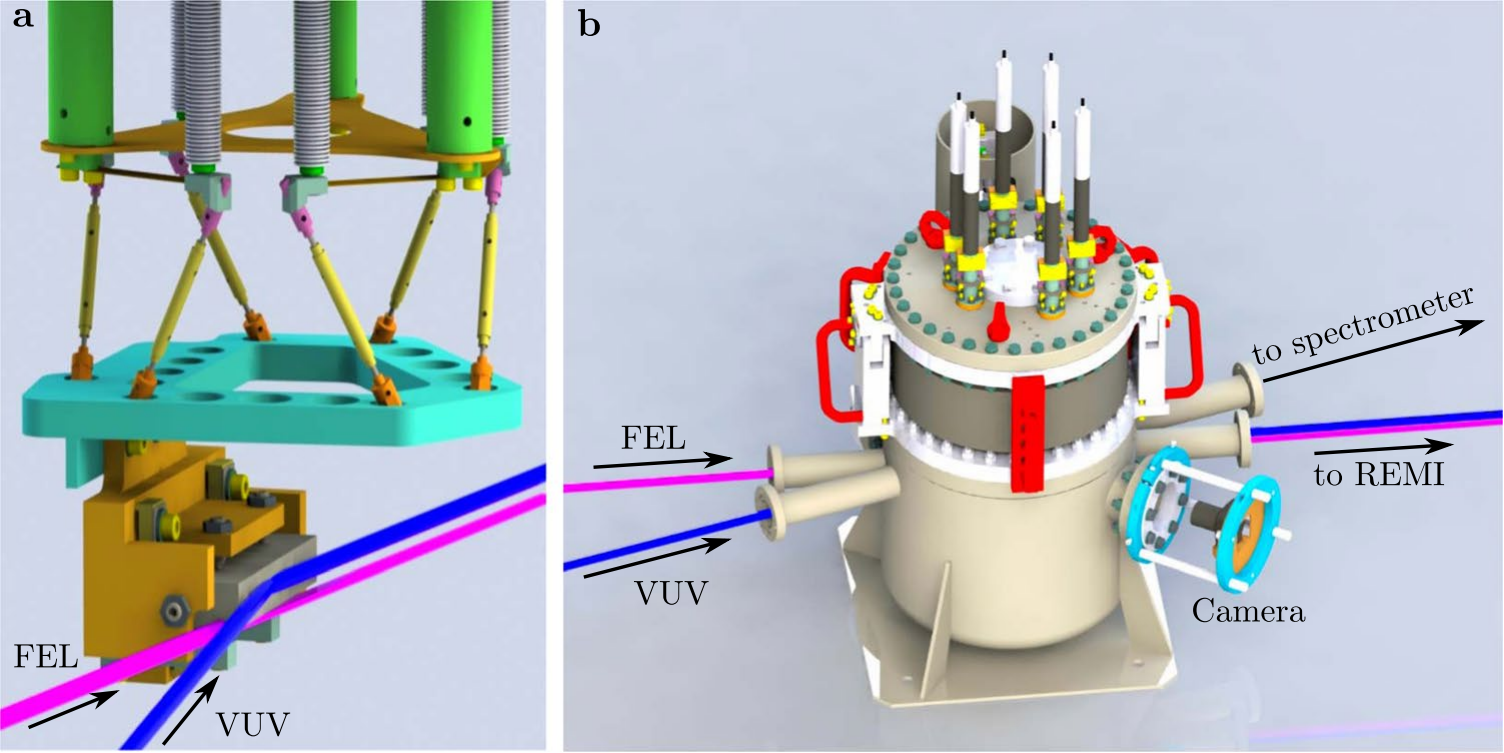
(**a**) In-coupling mirror assembly. The VUV beam is reflected on the hyperboloid mirror leaving the in-coupling chamber parallel to the FEL beam. (**b**) Chamber for the in-coupling of the VUV beam into the FEL beamline. The bellow on the top flange allows to remove the mirror from the beam path and to access the VUV spectrometer behind it. A camera system is used for imaging the mirror assembly inside the chamber.

Behind the filter wheel, the VUV beam enters the in-coupling chamber in the FEL beamline, where a carbon-coated hyperboloid mirror (designed by OptiXfab GmbH) can be inserted in the beam path. This mirror serves a dual purpose: collimation of the VUV radiation and in-coupling into the FEL beamline. In order to collimate the beam, the focal length of the hyperboloid mirror was chosen to be 1.5 m, equal to the distance from the harmonic generation point to the in-coupling position. A grazing angle of 8° allows the beam to enter the beamline parallel to the FEL (Fig. 4a). For this incidence angle, the carbon coating ensures a reflectivity close to 80% in the full spectral range of interest^24,28^. To align the VUV beam, a hexapod kinematics structure provides control of all the six degrees of freedom of the mirror position. The structure is connected to 6 linear motors placed on the in-coupling chamber top flange. The same design principle is used for the other XUV optics in the FL26 beamline^24^.

The reflected VUV beam propagates with a vertical shift of 7.5 mm with respect to the FEL. Due to this shift each beam reaches a different half of the following split-and-delay mirror and can be delayed with respect to each other. At the end of the beamline, both beams are focused, spatially overlapped, in the REMI end-station by the ellipsoidal mirror (see Fig. 2).

### In-line spectrometer

The top flange of the in-coupling chamber is mounted on a bellow which can be mechanically tilted up to 6° in order to move the mirror assembly completely out of the beam path. The reproducibility of the bellow position is sufficiently high that only fine adjustments of the in-coupling mirror, using the hexapod mechanism, are needed for re-aligning the VUV beam in REMI.

**Figure 5:**
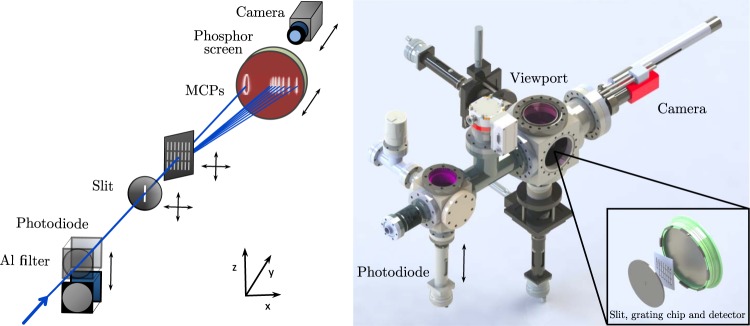
Schematics of the in-line spectrometer. The photodiode can be moved in and out of the beam path. A transmission grating separates spatially the spectral components of the harmonics spectrum, which are then detected by the MCP-phosphor assembly and observed by a camera placed behind the detector.

In this out-of-beam configuration (dashed IM outline in Fig. 2), the VUV beam reaches the in-line spectrometer (see Fig. 5). In the first part, a XUV photodiode (AXUV576, OptoDiode) is installed to measure the harmonic photon flux. The detector has been selected with a large active area to collect the full divergent harmonic beam. The remaining infrared light is blocked by a 0.1 μm thick Al filter directly in front of the photodiode. The entire assembly is placed on a linear feedthrough in order to be moved in and out of the beam path. Outside the vacuum system, the signal from the photodiode is amplified by a low-noise preamplifier (SR570, Stanford Research Systems) and then read out by an oscilloscope (DPO 5204, Tektronix).

In the second part, the spectral components of the VUV beam are spatially separated by a transmission grating, that allows for a very compact geometry suited to the limited working space in the experimental hall. We make use of a free-standing chip with multiple transmission gratings of different periods^29^. The 21 gratings with dimensions 1.0 × 4.0 mm^2^ and 1.0 mm/0.5 mm of spacing horizontally/vertically, are arranged on the chip in a two-dimensional array consisting of three lines and seven columns, with overall dimensions 15 × 15 mm^2^. The inner line structure of each grating, described by the number of periods per mm, determines the spectral analysis properties. A slit of 0.8 × 3.8 mm^2^ is placed in front of the chip to illuminate a specific grating. The slit and the chip can be moved independently in any direction using a combination of linear feedthroughs and 2D translation stages in order to optimize the alignment with respect to the VUV beam position. After the grating, the different spectral components are collected by a double microchannel plate (MCP) detector combined with a phosphor screen (DD 2561 ZV, Proxivision). The detector can be moved in the beam propagation direction with a 14 cm range (1 to 15 cm from the grating chip), depending on the required acceptance angle. A camera (acA2040–25 gm, Basler), placed outside the vacuum chamber, records the image from the detector.

### Vacuum conditions

Noble gas is inserted in the SIGC through a regulating valve (EVR 116, Pfeiffer). The pressure during operation varies between a few mbar and a few tens of mbar depending on the generation requirements and is actively controlled via a PID feedback with an oscillation of <0.2 mbar. The SIGC can be pumped down independently by a roughing pump. To avoid re-absorption of the generated VUV radiation, a turbo pump (HiPace700, Pfeiffer) on the HHG chamber removes most of the gas diffused through the hole in the Al plate, which acts like a first differential pumping stage. Thus, the pressure in the HHG chamber can be kept in the 10^−5^ mbar range, more than 5 orders of magnitude lower than the SIGC pressure. In order to reach the required operation conditions in the REMI chamber, several additional differential pumping stages are implemented. A tube with a 4 mm inner diameter separates the HHG chamber from the filter wheel section. Two turbo pumps (HiPace80 and HiPace30, Pfeiffer) placed before and after the filter wheel, maintain a pressure of 10^−7^ and 10^−8^ mbar respectively. At the input flange of the in-coupling chamber, a tube of inner diameter 10 mm is placed, which allows for a final pressure of <1 × 10^−9^ mbar at the intersection point in the FEL beamline. Additional differential pumping stages in the FEL beamline ensure an operation pressure in REMI at about 10^−11^ mbar.

## Results

While this work targets the perspective to address fundamental dynamics of matter by making use of combining complementary properties of FELs and laser-based HHG sources, the here presented work represents a feasibility study for implementing a laser-driven HHG source within an existing FEL beamline. To this end, our initial goals include the observation of harmonics with the present approach (in particular the SIGC) and the measurement of their basic parameters such as photon flux and spectral coverage, in order to evaluate the performance of such a VUV source directly at a FEL beamline.

### Harmonic generation

During commissioning, our initial target was to test all the necessary subsystems of the harmonics beamline. This test included the optical beam delivery, the gas supply, all vacuum systems and the spectrometer setup (see Fig. 2 and methods section). By using a SIGC setup for HHG^30,31^, the first necessary step was to enable the propagation of the radiation from the gas cell by drilling a hole in a thin metal plate. On the spectrometer unit, the spectrally resolved harmonic radiation was observed on the camera.

**Figure 6:**
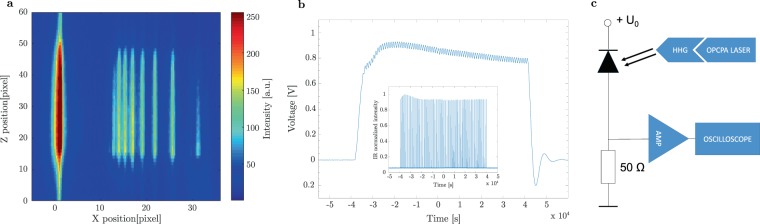
(**a**) Typical spectrum observed on the spectrometer detector. Left side: zero-order of the diffraction grating (1850 lines per mm), right side: spatially separated spectral components of the VUV signal. (**b**) Oscilloscope trace from the photodiode signal. Inset: laser burst pattern recorded by the optical laser diagnostics. (**c**) Electronic circuit for photodiode measurements.

Different resolutions are accessible by simply selecting a different grating on the grating chip and subsequently adapting the detector distance. Figure 6a shows a typical spectrum using a 1850 lines per mm grating. The bright line visible on the left side corresponds to the zero diffraction order of the grating, while on the right side the spectral components of the VUV beam are observed. The harmonic order decreases from left to right and the color-coding indicates the relative intensity of the different harmonic spectral lines.

Besides the spectral distribution, the most relevant parameter is the available VUV photon number. A typical recorded photodiode trace is presented in Fig. 6b, where the 80 pulses of the burst structure are resolved. In the inset, a typical laser burst pattern, as recorded by the optical laser diagnostics, is shown. The photodiode signal’s slow modulation does not match exactly the laser burst envelope shape, which we attribute to a combination of the nonlinearity of the HHG process and the electronic circuit response. By taking into account the circuit properties as well as the diode response, the pulse energy of the VUV radiation can be calculated from the integral of the oscilloscope trace. We estimated a maximum VUV pulse energy from the source of 0.1 nJ (conversion efficiency of 10^−7^), after correcting for two aluminium filters used in order to suppress unwanted IR stray light. Figure 6c shows the electronic setup for the absolute photon count measurements of the harmonics beamline.

**Figure 7:**
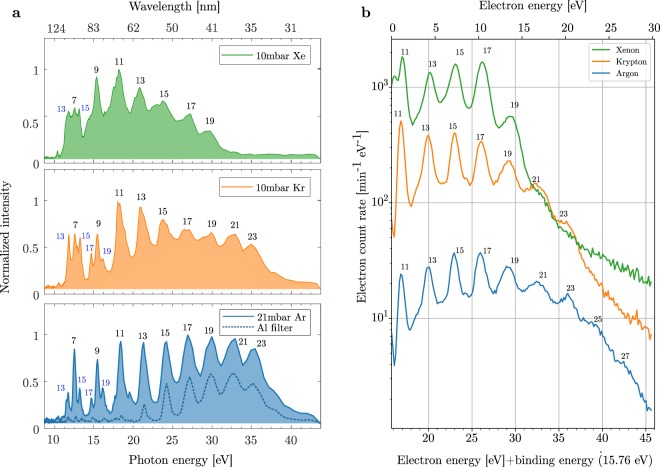
(**a**) Harmonics spectra from the VUV spectrometer, generated with 10 mbar of Xe (top), 10 mbar of Kr (center) and 21 mbar of Ar (bottom). The black and blue numbers indicate the harmonic orders for the grating’s first and second order of diffraction respectively. (**b**) Electron energy spectrum for photo-ionization of Ar with harmonics generated in Xe (green), Kr (orange), Ar (blue). In these measurements a 4 mm aperture was used to suppress the IR radiation. The measured energy is not calibrated to a reference, but calculated from the measured magnetic flux and consistent calibration of the electric field strength in REMI.

In Fig. 7a we show a comparison of HHG spectra from different noble gas media with a 1850 lines per mm grating. With a pressure of 21 mbar of Ar in the SIGC, we clearly observed up to the 23^rd^ harmonic order, which corresponds to a maximum photon energy of 36 eV. The secondary peaks around the 7^th^ and 9^th^ order correspond to the grating’s second order of diffraction. The dotted line shows the spectrum after the insertion of a 0.1 μm thick aluminium filter in the beam path. The filter suppresses the fundamental (only observed in the zero order of diffraction in the present configuration) and the low order harmonics, while the high order harmonics are transmitted. Figure 7a demonstrates that different bandwidths can be tailored by either changing the generation gas or/and introducing an aluminium filter as shown in the example of Ar.

### Reaction microscope

The REMI detection scheme allows to measure ions and electrons in coincidence by reconstructing their 3D momentum with $4\pi$ acceptance angle. The coincidence detection is achieved by guiding the fragments onto time and position sensitive detectors via electric and magnetic fields. For more information on the capabilities of the REMI end-station at FL26 see Schmid *et al*.^24^.

The FEL beamline features an array of apertures of different diameter after the VUV in-coupling chamber and before an in-line split-and-delay mirror (SDM) followed by a focusing mirror (FM) (see Fig. 2). After being coupled into the FEL beamline, the VUV beam is reflected on the upper part of the planar SDM and focused into a supersonic gas jet target in REMI by the off-axis ellipsoidal mirror. The three beamline mirrors’ reflectivity (80% each) adds up to 51% without using any filter.

For the commissioning experiments described here, an argon jet was used as target in REMI. An Al filter was installed in the VUV filter wheel with the purpose of blocking the fundamental wavelength. A diameter of 4 mm for the beamline aperture was chosen to closely match the diameter of the VUV beam. Using only the aperture was a trade-off between blocking most of the more divergent fundamental light and transmitting as much VUV light as possible. The Ar ion rate was measured for three different gases in the SIGC, with and without the Al filter and the aperture in the beam path. An overview of the measured Ar ion rates and estimated photon numbers is listed in Table 1 for several combinations.

**Table 1:** (a) Argon photoionization rate per pulse. The rate was measured for different combinations of transmission optics and VUV source gas. (b) Photons per pulse, estimated from photoion rates, electron energy spectra and tabulated photoionization cross-sections at the harmonic photon energies^42^.

(a)				(b)
filter aperture	Al 0.1 μm no aperture	no filter aperture 4 mm	no filter no aperture	no filter aperture 4 mm
Xe	0.075	0.405	0.504	3.1 × 10^6^
Kr	/	0.110	/	1.1 × 10^6^
Ar	0.005	0.012	0.032	0.27 × 10^6^

For both Xe and Ar as generation gases the maximum ion rate was reached without the Al filter and without the aperture. Using the Al filter alone reduces the ion rate for both gases more than using just the aperture. A higher ionization rate was measured for Xe compared to Ar for all settings. The rate for Kr was between the rates for the other two gases with the same filter/aperture combination.

In terms of the absolute number of photons per pulse measured in REMI for the case of the 4 mm aperture in the beam path (Table 1b), Xe as generating medium is clearly the most efficient gas. The number of 3.1 × 10^6^ photons/pulse corresponds to 0.01 nJ per pulse. By taking into account that 51% of the generated photons reaches the interaction region and that photon energies below the 11^th^ harmonic order are not sufficient for Ar ionization by single-photon absorption (ionization potential of Ar: 15.76 eV), the maximum energy per pulse estimated from the REMI measurements seems consistent with the photodiode estimations within the error limits.

**Figure 8:**
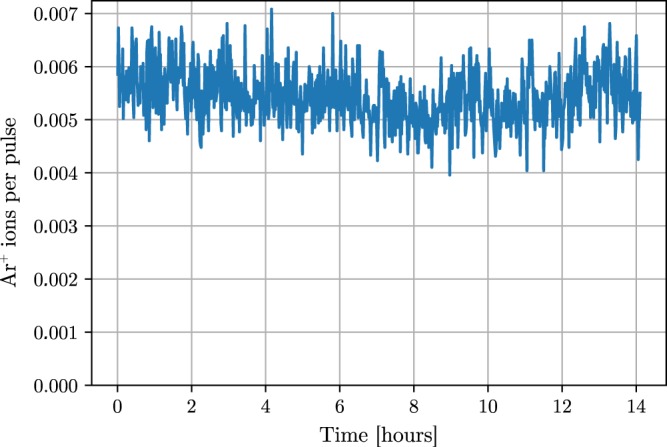
Ar ion rate per pulse for a long run (14 hours) of continuous data acquisition. The rate was averaged over 1 min. The harmonics were generated in Ar and the beam was filtered with a 0.1 μm thick Al filter.

The overall stability of the harmonic source in terms of beam pointing and flux can be evaluated by the ionization rate in REMI. Figure 8 shows the event rate of singly ionized argon during a 14-hours-long measurement. The rate varies by less than 20% over the whole time. This measurement demonstrates that the VUV source is sufficiently stable for long experiments in combination with the FEL.

**Figure 9:**
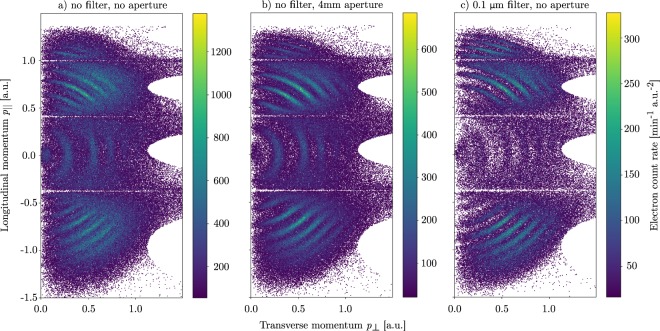
Histograms of the argon photo-electron momentum in transverse and longitudinal direction to the spectrometer axis (coincides with the VUV polarization axis). Half circles in this momentum plane correspond to constant energy of the electron. Harmonics were generated in 40 mbar of Ar in the SIGC. In the beamline for (**a**) no filter, for (**b**) a 4 mm aperture and for (**c**) a 0.1 μm aluminium filter was inserted.

The measured electron momentum distributions (see Fig. 9) show several lines of constant kinetic energy corresponding to the excess harmonics photon energies with respect to the ionization potential. The 2D distributions are shown as a function of the longitudinal and transverse momentum, *p*_||_ and ${p}_{\perp }$, with respect to the polarization axis in cylindrical coordinates. Note that the count rate vanishes towards zero transverse momentum due to the decreasing solid angle. Therefore maxima in count rate appear only close to zero transverse momentum although emission in the polarization direction (vertical) is preferred. When the fundamental IR wavelength is not suppressed, it modulates the electron energy and sidebands at the energies of even harmonics appear (Fig. 9a). Sidebands for emission in transverse direction are not observed. The modulation is only present if the electron is emitted close to the polarization direction. With the Al filter (Fig. 9c), only odd harmonics appear in the electron spectrum, but the absolute rate is significantly reduced. Sidebands are also strongly suppressed when using a 4 mm aperture, which blocks mainly the more divergent IR beam. This effect can be seen in Fig. 9b, where the sideband contributions almost vanish.

Figure 7b presents the electron rates with respect to the sum of the electron and the Ar binding energy (15.76 eV) for the three different generation gases. The same harmonic lines as those observed in the spectrometer (Fig. 7a) are also clearly visible in the electron spectra, starting from the first line (11^th^ harmonic) above the ionization threshold.

It should be noted that, although the 4 mm aperture is a good compromise between the options of a 0.1 μm thick Al filter and no filter in the beam path in order to avoid the IR contamination and to maintain the VUV photon flux as high as possible, it cannot be used during VUV-XUV pump-probe experiments in the current configuration. Due to the difference in height, when the 4 mm aperture is in use for the VUV beam, the FEL is blocked by the rim of the apertures array. The two beams can simultaneously pass only through one large aperture (see inset of Fig. 2). To overcome this problem, we plan to decouple the size control of the two beams by using an already existing apertures array before the in-coupling chamber for the FEL and installing apertures of different sizes in the filter wheel for the VUV beam.

## Discussion

We demonstrated the first realization of a permanent VUV beamline installed as pump-probe source at a free-electron laser. Our findings show that the generation of tunable radiation in a spectral window from 10 to 40 eV is possible with a flux of about 80 nJ/s on a daily basis, still leaving room for improvements. The harmonic beamline is fully synchronized with the burst-mode of the free-electron laser FLASH. During commissioning of the beamline, stable operation conditions could be demonstrated for at least 14 hours, allowing extended acquisition times. First commissioning tests were performed using the example of Ar ionization by VUV laser pulses recorded via photoion rates measurements and electron momentum imaging using REMI. Future experiments could include, for example, non-sequential ionization processes with low probability and coincidence detection enabling so-called kinematically complete experiments^32^. Moreover, the already demonstrated measurements of ions and electrons within REMI displays the potential of the harmonic beamline with regard to experiments involving versatile detection approaches in combination with the FEL.

It should be noted that we see a high potential for future improvements of this HHG beamline. Foremost, the generated harmonic photon flux needs to be further optimized to the current state-of-the-art, which so far was not possible due to very restricted commissioning time. Improving the HHG emission towards higher conversion efficiency requires the optimization of both the microscopic and macroscopic properties of the process. HHG signal optimization will be performed by scanning the main parameters that contribute to phase-matching, like driving pulse intensity, gas pressure and focusing conditions. In addition, the possibility to use the second and third harmonic of the fundamental laser as HHG driving field can offer new capabilities, including a much higher photon flux^33^ and a larger spectral separation between adjacent harmonics.

Spectral control over the generated HHG radiation can be offered by different single or multiple spectral filters. So far, we demonstrated a spectral filtering of the generated VUV radiation selecting a bandwidth of about 20 eV corresponding to harmonics order from 13 to 25 with a 0.1 μm thick aluminium filter. More narrowband VUV emission can be obtained by the use of various available filter materials. For example, In, Sn and Ge filters can provide transmission windows of about 10 eV over almost the full range of the photon energies generated here. Moreover, working at the second or third harmonic of the fundamental wavelength enables more efficient notch filtering of selected harmonic lines. In addition, multi-layer mirrors can support the selection of individual harmonic orders^34^.

In summary, the reported VUV beamline is the first permanent HHG-based installation at a large-scale FEL facility. On a day-to-day basis, the source is capable of providing an efficiency of 10^−7^ with stable operating conditions for more than 14 hours. The harmonic source is tunable in bandwidth and fully synchronized to the burst-mode structure of the FLASH facility. The measured harmonic flux allows for multi-particle coincidence spectroscopy on gas-phase samples, setting the stage for XUV-VUV pump-probe investigation of atoms, molecules and clusters on femtosecond timescales.

## Data Availability

The authors declare that the data generated and analyzed presented in the manuscript are available from the corresponding author upon reasonable request.
